# Tissue-specific requirements for specific domains in the FERM protein Moe/Epb4.1l5 during early zebrafish development

**DOI:** 10.1186/1471-213X-8-3

**Published:** 2008-01-11

**Authors:** Arne K Christensen, Abbie M Jensen

**Affiliations:** 1 Department of Biology and the Molecular and Cellular Biology Program, University of Massachusetts, Amherst, MA, 01003, USA

## Abstract

**Background:**

The FERM domain containing protein Mosaic Eyes (Moe) interacts with Crumbs proteins, which are important regulators of apical identity and size. In zebrafish, loss-of-function mutations in *moe *result in defects in brain ventricle formation, retinal pigmented epithelium and neural retinal development, pericardial edema, and tail curvature. In humans and mice, there are two major alternately spliced isoforms of the Moe orthologue, Erythrocyte Protein Band 4.1-Like 5 (Epb4.1l5), which we have named Epb4.1l5^long ^and Epb4.1l5^short^, that differ after the FERM domain. Interestingly, Moe and both Epb4.1l5 isoforms have a putative C' terminal Type-I PDZ-Binding Domain (PBD). We previously showed that the N' terminal FERM domain in Moe directly mediates interactions with Crumbs proteins and Nagie oko (Nok) in zebrafish, but the function of the C'terminal half of Moe/Epb4.1l5 has not yet been examined.

**Results:**

To define functionally important domains in zebrafish Moe and murine Epb4.1l5, we tested whether injection of mRNAs encoding these proteins could rescue defects in zebrafish *moe*^- ^embryos. Injection of either *moe *or *epb4.1l5*^*long *^mRNA, but not *epb4.1l5*^*short *^mRNA, could rescue *moe*^- ^embryonic defects. We also tested whether mRNA encoding C' terminal truncations of Epb4.1l5^long ^or chimeric constructs with reciprocal swaps of the isoform-specific PBDs could rescue *moe*^- ^defects. We found that injection of the Epb4.1l5^short ^chimera (Epb4.1l5^short+long_PBD^), containing the PBD from Epb4.1l5^long^, could rescue retinal and RPE defects in *moe*^- ^mutants, but not brain ventricle formation. Injection of the Epb4.1l5^long ^chimera (Epb4.1l5^long+short_PBD^), containing the PBD from Epb4.1l5^short^, rescued retinal defects, and to a large extent rescued RPE integrity. The only construct that caused a dominant phenotype in wild-type embryos, was Epb4.1l5^long+short_PBD^, which caused brain ventricle defects and edema that were similar to those observed in *moe*^- ^mutants. Lastly, the morphology of rod photoreceptors in *moe*^- ^mutants where embryonic defects were rescued by *moe *or *epb4.1l5*^*long *^mRNA injection is abnormal and their outer segments are larger than normal.

**Conclusion:**

Taken together, the data reveal tissue specificity for the function of the PBD in Epb4.1l5^long^, and suggest that additional C' terminal sequences are important for zebrafish retinal development. Additionally, our data provide further evidence that Moe is a negative regulator of rod outer segment size.

## Background

The mechanisms underlying the acquisition and maintenance of apical cell polarity are beginning to be understood and the importance of cell polarity in development is now widely appreciated. Drosophila Crumbs (Crb) and vertebrate Crumbs orthologues are important determinants of apical polarity and are critical for epithelial morphology [1-4]. The establishment of cell polarity within the developing retinal neuroepithelium is crucial for normal retinal development, as zebrafish with loss-of-function mutations in the polarity determinants *aPKCλ/heart and soul *(*has*), *pals1/mpp5/nagie oko *(*nok*), *crb2a/oko meduzy *(*ome*), and *mosaic eyes *(*moe*), fail to properly form cell-specific laminae [4-9]. In addition, ablation of *aPKCλ *in differentiating photoreceptors in a conditional knockout mouse results in a loss of retinal lamination [10].

Crumbs proteins are also important for normal photoreceptor morphogenesis and zonula adherens/adherens junction formation and/or maintenance in Drosophila [11-14]. In humans, mutations in the *CRUMBS HOMOLOGUE-1 *(*CRB1*) gene cause retinal degeneration diseases [15-19]. Mouse models lacking functional Crb1 exhibit a compromised outer limiting membrane (OLM) in the retina and defects in photoreceptor morphology [13,14]. Furthermore, data from our lab and others have implicated the Crumbs complex as a key regulator of apical membrane size in photoreceptors [4,11,12,20,21].

The *moe *mutant was discovered in a zebrafish mutagenesis screen, and the *moe *mutations affect retinal lamination, brain ventricle formation, and heart and body morphology [7,22]. Orthologues have been identified in Drosophila (Yurt) and mammals (Erythrocyte Protein Band 4.1-Like 5, Epb4.1l5) [22,23]. The *yurt *and *epb4.1l5 *locus encode four and two isoforms respectively [20,24]. We and our colleagues have shown that Moe and Moe orthologues form a complex with Crumbs proteins that is mediated by the FERM domain, and this interaction is important for Crumbs protein function [20,21]. The mouse mutant *lulu *has a null-allele mutation in *epb4.1l5*, and has defects in the epithelial-mesenchymal transition in cells at the primitive streak and abnormal neural plate morphology that is accompanied by defects in the actin-cytoskeleton [24]. In this study we use a comparative genomic and proteomic approach to identify functionally important sequences within Moe and Epb4.1l5 by testing whether injection of mRNA encoding the long and short isoforms of Epb4.1l5 (Epb4.1l5^long ^and Epb4.1l5^short^) can functionally substitute for *moe *function in zebrafish. We further investigate the role of Moe within different tissues by defining what Epb4.1l5 domains are necessary to rescue distinct *moe*^- ^defects. Lastly, we report the histological and morphological consequences of losing Epb4.1l5^long ^protein in the rescued zebrafish retina after the depletion of rescue construct.

## Results

### Injection of *moe *mRNA rescues embryonic defects in *moe*^- ^mutants

**Figure 1:**
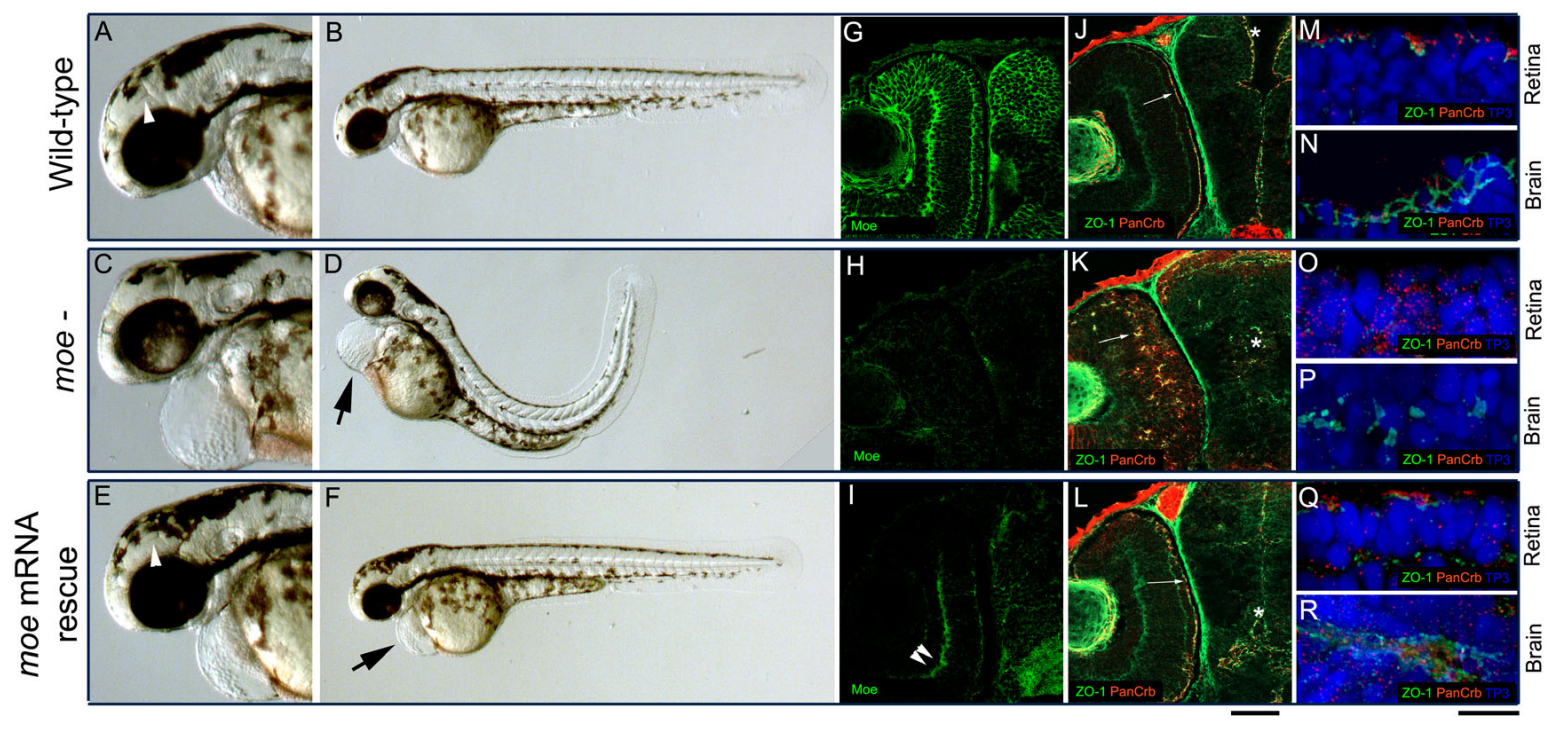
**Injection of *moe *mRNA rescues defects in *moe*^- ^embryos**. (A-B) At 60 hpf, in wild-type embryos, the floor of the diencephalic ventricle is visible (A, white arrow head), the RPE is uniform, and the body axis is straight. (C, D) In *moe*^- ^embryos, the ventricles are small or absent, the RPE is patchy, the tail curves and there is pericardial edema (D, arrow). (E, F) In *moe*^- ^embryos injected with *moe *mRNA, the floor of the diencephalic ventricle is visible (E, white arrow head), the RPE is uniform, and the body axis is straight but mild pericardial edema persists (F, arrow). Anti-Moe labeling of 60 hpf wild-type embryos (G), *moe*^- ^embryos (H), and *moe*^- ^embryos injected with *moe *mRNA (I): the plexiform labeling in *moe*^- ^embryos injected with *moe *mRNA (I, double arrowheads) is largely background. Adherens junctions (ZO-1, green) and panCrb labeling (red) are apically localized at the retina (arrow) and brain ventricle surface (asterix) in wild-type (J) and *moe*^- ^embryos injected with *moe *mRNA (L), but are ectopically localized within the developing eye (arrow) and at the presumptive brain midline in *moe*^- ^mutants with abnormal ventricle formation (asterix, K). High magnification confocal z-projections of TO-PRO-3 nuclear staining, and ZO-1 and panCrb labeling in the retina and brain in wild-type (M, N), *moe*^- ^(O, P), and *moe*^- ^embryos injected with *moe *mRNA (Q, R) at 60 hpf. (G, J, H, K, I and L are all single confocal z-sections). Scale bars, 50 μm (G-L), 10 μm (M-R).

We tested whether injecting *moe *mRNA into *moe*^- ^embryos could rescue embryonic and early larval defects. We found that injection of wild-type *moe *mRNA into *moe*^- ^mutant embryos at the 1–4 cell-stage rescued brain ventricle formation, retinal pigmented epithelial (RPE) integrity, and retinal neural epithelial integrity and straightened the tail at 60 hours post fertilization (hpf) (Figure 1). We observed no abnormalities in injected wild-type embryos (or larvae). Pericardial edema in *moe*^- ^mutants was only partially rescued by *moe *mRNA injection (Figure 1D, F, arrow). The remaining pericardial edema was a convenient marker, however, and made it possible to easily distinguish between wild-type larvae and rescued *moe*^- ^mutants, but we also confirmed that embryos were *moe*^- ^mutants by labeling with anti-Moe antibodies at 60 hpf and comparing labeling to wildtypes and uninjected *moe*^- ^mutants (Figure 1G, H, I). In *moe *mRNA injected *moe*^- ^mutants very little anti-Moe labeling was observed except background (Figure 1I, double arrowheads). The weak anti-Moe labeling in *moe *mRNA injected *moe*^- ^mutants suggests very little Moe protein encoded by the injected mRNA remains at 60 hpf.

We have shown that Moe interacts with Crumbs proteins, which are important apical polarity determinants and that *moe *loss-of-function results in a failure to localize Crb2a and the junctional protein ZO-1 at the apical surface of the retina and brain [21,22]. We examined whether injection of *moe *mRNA into *moe*^- ^mutants could rescue the apical localization of Crumbs proteins and ZO-1 in the retina and brain. In order to examine Crumbs proteins in zebrafish, we used an antibody we raised against the highly conserved C' terminal peptide and because this antibody recognizes all zebrafish Crumbs proteins by western blot (data not shown) we call this antibody a panCrb antibody. In wild-type embryos at 60 hpf, anti-panCrb and anti-ZO-1 labeling localize to the apical/ventricle surface in the brain, and the apical surface and the newly forming outer limiting membrane in the retina (Figure 1J, M, N). In *moe*^- ^mutants, brain ventricles fail to form properly, and panCrb and ZO-1 fail to localize to the apical surfaces in the brain and retina (Figure 1K, O, P). Injection of *moe *mRNA into *moe*^- ^mutants leads to the apical relocalization of Crumbs proteins and ZO-1 in the retina and brain (Figure 1L, Q, R).

### Conservation between Moe and mouse Epb4.1l5

**Figure 2:**
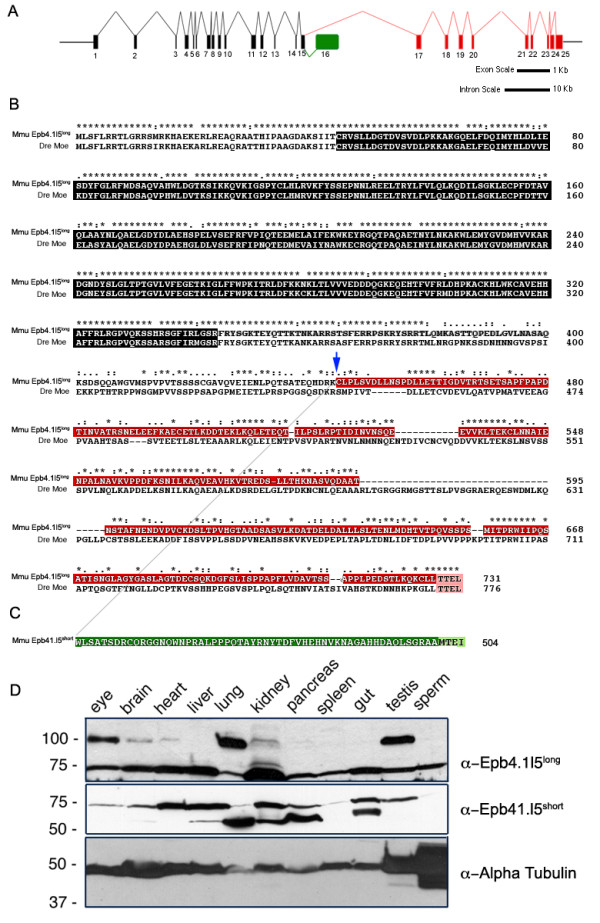
**Genomic structure of the mouse *epb4.1l5 *locus and expression of its two major splice isoforms**. (A) Diagram of the inton/exon structure of the *Mus musculus epb4.1l5 *locus. Exons that are common to both isoforms are black, the exons unique to *epb4.1l5*^*long *^are indicated in red and the unique exon in *epb4.1l5*^*short *^is indicated in green. Bars represent 1 kb and 10 kb scales for exon and intron lengths, respectively. (B) ClustalX alignment of mouse Epb4.1l5^long ^and zebrafish Moe. Ymo1^long ^and Moe share a high degree of homology within the FERM domain (black). Ymo1^long ^and Ymo1^short ^are identical up to Lysine 444 (blue arrow) and then alternately spliced into the long (red) and short (green) isoforms. Moe and Epb4.1l5^long^, and Epb4.1l5^short ^have predicted C'terminal PDZ-binding domains (Pink [TTEL]) and (light green [MTEI]). (*) identical, (:) highly conserved, (.) moderately conserved. (C) Western analysis of mouse tissues with antibodies raised against the unique C'terminal sequences of Epb4.1l5^long ^and Epb4.1l5^short^. Two bands are immunoreactive with the anti-Epb4.1l5^long ^antibody, one migrates at the expected molecular weight of Epb4.1l5^long ^(100 kDa) and is present in the eye, brain, heart, lung, kidney, and testis. An additional band migrates at 75 kDa, which is probably non-specific. Two bands are recognized by the affinity purified Epb4.1l5^short ^antibody. A lower band migrates at the predicted molecular weight of 56 kDa and is present in brain, liver, lung, kidney, pancreas, and gut. A second band with a broad expression pattern migrates at approximately 75 kDa. Anti-α-Tubulin was used as a loading control.

To help identify functionally important domains in the Moe and Epb4.1l5 proteins, we first compared their sequences (Figure 2). The mammalian *epb4.1l5 *locus encodes two major splice isoforms that are represented by ESTs in both the human and mouse databases, which we term Epb4.1l5^short ^and Epb4.1l5^long^. We provide the exon/intron structure of the mouse *epb4.1l5 *locus that has 25 exons: Epb4.1l5^short ^is encoded by exons 1–16 and Epb4.1l5^long ^by exons 1–15 and 17–25 (Figure 2A). We have not found a zebrafish transcript that encodes a protein similar to Epb4.1l5^short^.

A comparison between Moe and mouse Epb4.1l5^long ^shows very strong homology in the FERM domain (88% identity), and there is also strong conservation flanking the FERM domain (44 amino acids preceding the FERM domain and about 35 amino acids after) as well as additional islands of strong conservation, notably a class I PDZ-binding domain (PBD) at the C' terminus of both proteins (Figure 2B; [24,25]). Homology between Moe and Epb4.1l5^short ^ends at amino acid 444 (Figure 2C, blue arrow), after which Epb4.1l5^short ^has 60 unique amino acids (Figure 2C). Epb4.1l5^short ^is predicted to be 56 kDa, and interestingly also has a predicted binding motif for a class I PDZ domain at its C' terminus (Figure 2C).

We raised isoform-specific antibodies against the unique C' terminal sequences of the long and short isoform of Epb4.1l5 and used them for western analysis of mouse tissue. We observed two bands that were immunoreactive with Epb4.15^long ^anti-sera. A protein that migrated at approximately 100 kDa was present in eye, brain, heart, lung, kidney, and testis tissue (Figure 2D). There was an additional protein recognized in all tissues at 75 kDa, which is probably non-specific reactivity. Two proteins were detected with anti-Epb4.1l5^short ^affinity-purified antibody. One protein migrated at the expected molecular weight of 56 kDa and was present in brain, liver, lung, kidney, pancreas, and gut. A second protein migrating at approximately 75 kDa was broadly expressed. This higher molecular weight protein may represent a post-translationally modified form of Epb4.1l5^short ^or more likely is non-specific reactivity (Figure 2D).

### Functional comparative genomics reveals important domains in Moe/Epb4.1l5

**Figure 3:**
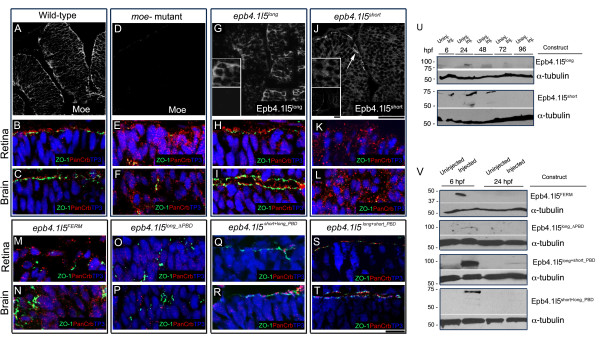
**Both the PDZ-binding domain and unique sequences in Epb4.1l5^long ^required for rescue of *moe*^- ^mutant defects**. (A-C) At 30 hpf in wild-type embryos, Moe localizes cortically in brain and retinal neuroepithelial cells and is concentrated at the apical surface (A) and ZO-1 (green) and panCrb (red) localize to the apical surface of the retina (B) and brain (C). (D-F) At 30 hpf in *moe*^- ^embryos, there is no Moe labeling (D) and ZO-1 (green) and panCrb (red) fail to localize to the apical surface of the retina (E) and brain (F). (G) At 30 hpf in *moe*^- ^embryos injected with *epb4.1l5*^*long *^mRNA, Epb4.1l5^long ^immunoreactivity is cortically localized in most retinal and brain neuroepithelial cells and ZO-1 (green) and panCrb (red) localize to the apical surface in the retina (H) and brain (I). Upper inset (G), magnified section of anti-Epb4.1l5^long ^labeling and lower inset, uninjected *moe*^- ^embryos shows no labeling with anti-Epb4.1l5^long^. (J) At 30 hpf in *moe*^- ^mutants injected with *epb4.1l5*^*short *^mRNA, anti-Epb4.1l5^short ^is cytoplasmically localized and ZO-1 (green) and panCrb (red) do not localize to the apical surface in *moe*^- ^retina (K) and brain (L). Upper inset (G), magnified section of anti-Epb4.1l5^short ^labeling and lower inset, uninjected *moe*^- ^embryos shows no labeling with anti-Epb4.1l5^short^. (M, N) At 30 hpf in *moe*^- ^mutants injected with *myc-epb4.1l5*^*FERM *^mRNA, ZO-1 (green) and panCrb (red) do not localize to the apical surface in *moe*^- ^retina (M) and brain (N). (O, P) At 30 hpf in *moe*^- ^mutants injected with *myc-epb4.1l5*^*long_ΔPBD *^mRNA, ZO-1 (green) and panCrb (red) do not localize to the apical surface in *moe*^- ^retina (O) and brain (P). (Q, R) At 30 hpf in *moe*^- ^mutants injected with *myc-epb4.1l5*^*short*+long_PBD ^mRNA, ZO-1 (green) and panCrb (red) localize to the apical surface in *moe*^- ^retina (Q) and brain (R). (S, T) At 30 hpf in *moe*^- ^mutants injected with *myc-epb4.1l5*^*long+short_PBD *^mRNA, ZO-1 (green) and panCrb (red) localize to the apical surface in *moe*^- ^retina (S) and brain (T). (U) Western analysis of zebrafish embryos injected with *epb4.1l5*^*long *^and *epb4.1l5*^*short *^mRNA were tested for expression of protein product with isoform-specific antibodies at time points from 6 hpf to 96 hpf. (V) Western analysis of zebrafish embryos injected with *epb4.1l5*^*FERM *^(anti-Myc), *epb4.1l5*^*long_ΔPBD *^(anti-Epb4.1l5^long^), *epb4.1l5*^*short+long_PBD *^(anti-Myc), *epb4.1l5*^*long+short_PBD *^(anti-Epb4.1l5^long^). Blots stripped and reprobed with Anti-α-Tubulin as a loading control. Scale bars, 10 μm (A, D, G, J), 50 μm (lower insets in G, J), 10 μm (remaining panels). (A-T), single confocal z-sections.

To identify functionally important sequences in the Moe and orthologous protein Epb41.l5, we tested whether injection of mouse *epb4.1l5*^*long *^mRNA could substitute for *moe *and rescue *moe*^- ^mutant defects. We injected *epb4.1l5*^*long *^mRNA into 1–4 cell *moe*^- ^embryos and found that it rescued brain ventricle formation, retinal pigmented epithelial integrity, and retinal lamination and straightened the tail like injection of *moe *mRNA (Table 1 and data not shown). Injection of *epb4.1l5*^*long *^also rescued apical localization of ZO-1 and anti-panCrb labeling in the retina and brain (Figure 3H, I). Using antibodies we raised against the unique sequence in Epb4.1l5^long^, we found that Epb4.1l5^long ^protein in mRNA injected *moe*^- ^mutants localized cortically like endogenous Moe in wild-type embryos (Figure 3A, G). Because all the proteins shown to interact with Moe do so through its FERM domain [21], we tested whether injection of *epb4.1l5 *mRNA encoding a myc-tagged FERM domain (amino acids 1–346, Epb4.1l5^FERM^) could rescue *moe*^- ^mutant defects like full length *moe *and *epb4.1l5*^*long *^mRNA injection. Injection of *epb4.1l5*^*FERM *^mRNA at the 1–4 cell stage failed to rescue the defects in *moe*^- ^mutants (data not shown) and did not lead to apical relocalization of ZO-1 and anti-panCrb labeling (Figure 3M, N). We also ruled out the possibility that the N' terminal myc-tag interfered with protein function by showing that Epb4.1l5^long ^myc-tagged at its N' terminus was still able to rescue *moe*^- ^mutant defects as well as untagged Epb4.1l5^long ^(data not shown)

**Table 1:** Quantitative assessment of phenotypic rescue of *moe*^- ^mutant defects by injection of *moe *and *epb4.1l5 *mRNA constructs.

Injected mRNA Construct	Genetic Background	% Edema	% Brain Ventricle Defects	% RPE Defects	Apical ZO-1/panCrb	Retinal Lamination
-	*wt *control	0% (0/81)	0% (0/81)	0% (0/81)	Yes	Yes
-	*moe*^+/- ^incross	25% (22/88)	25% (22/88)	25% (22/88)	No	No
*moe*	*moe*^+/- ^incross	24% (8/34)	0% (0/34)	0% (0/34)	Yes	Yes
*epb4.1l5*^*long*^	*moe*^+/- ^incross	25% (6/24)	0% (0/24)	0% (0/24)	Yes	Yes
*epb4.1l5*^*short*^	*moe*^+/- ^incross	27% (10/37)	27% (10/37)	24% (9/37)	No	No
*epb4.1l5*^*FERM*^	*moe*^+/- ^incross	24% (4/17)	24% (4/17)	24% (4/17)	No	No
*epb4.1l5*^*long_ΔPBD*^	*moe*^+/- ^incross	26% (30/86)	26% (30/86)	26% (30/86)	No	No
*epb4.1l5*^*short+long_PBD*^	*moe*^+/- ^incross	19% (9/48)	23% (11/48)	0% (0/48)	Yes	Yes
*epb4.1l5*^*long+short_PBD*^	*moe*^+/- ^incross	61% (20/47)	96% (54/56)	1% (1/88)*	Yes	Yes
*epb4.1l5*^*long*^	*wt*	0% (0/16)	0% (0/16)	0% (0/16)	NA	NA
*epb4.1l5*^*long+short_PBD*^	*wt*	20% (7/35)	42% (32/63)	3% (1/35)	NA	NA

**Figure 4:**
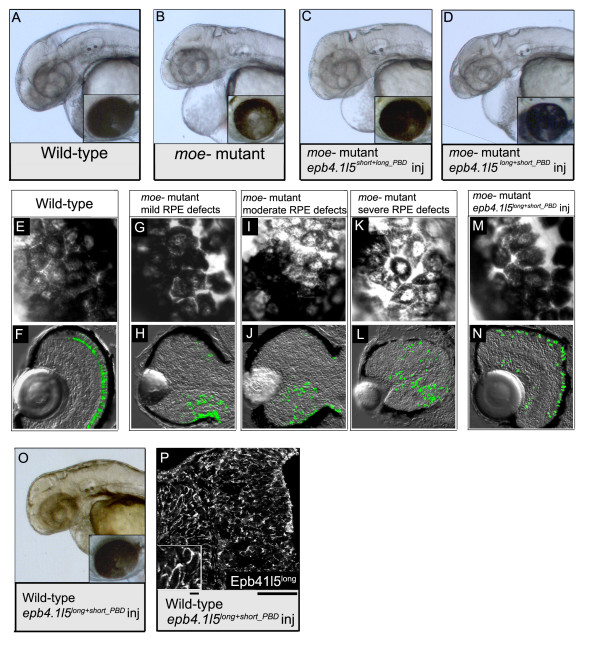
**Morphological rescue of some *moe*^- ^embryonic defects by injection of the *epb4.1l5 *constructs**. (A) In wild-type embryos at 60 hpf, brain ventricles are visible, and the RPE is uniform (inset). (B) In *moe*^- ^embryos, brain ventricles are reduced in size or absent, pericaridal edema is pronounced, and the RPE is patchy (inset). (C) In *moe*^- ^embryos injected with *epb4.1l5*^*short+long_PBD *^mRNA, brain ventricles are reduced in size or absent, pericaridal edema is pronounced, but the RPE is normal (inset). (D) *moe*^- ^embryos injected with *epb4.1l5*^*long+short_PBD *^mRNA, brain ventricles are absent or absent and pericardial edema is pronounced, and RPE defects are milder than those in uninjected *moe*^- ^embryos. (E) A magnified view of the RPE of a 60 hpf wild-type embryo shows that it is uniform and the cells are confluent. (F) In a wild-type retina at 4 dpf, GFP^+ ^rods localize next to the RPE and lamination is apparent. In 60 hpf *moe*^- ^mutants, the integrity of the RPE varies from mild (G), to moderate (I), to severe (H). However, GFP^+ ^rods are mislocalized in all *moe*^- ^mutants 4 dpf (H, J, L). The integrity of the RPE is improved and nearly normal in a 60 hpf *moe*^- ^mutant injected with *epb4.1l5*^*long+short_PBD *^mRNA (M) and most GFP^+ ^rods are adjacent to the RPE (N). (O) A wild-type embryo injected with *epb4.1l5*^*long+short_PBD *^showing brain ventricles that are reduced or absent. (P) At 30 hpf Epb4.1l5^long+short_PBD ^is cortically localized, upper inset is a 2× magnification of Epb4.1l5^long+short_PBD ^localization. Scale bars, 10 μm (F), 50 μm (lower insets in F).

Wild-type embryos or embryos from heterozygous *moe*^+/- ^incrosses were injected with the indicated mRNA constructs and scored based on pericardial edema, brain ventricle defects, RPE defects, apical localization of ZO-1 or Crb2a at 30 hpf, and retinal lamination at 5 dpf. Phenotypes that were not analyzed are indicated with an NA. * See Figure 4M, N and text.

We next tested whether injection of mRNA encoding Epb4.1l5^short ^could rescue *moe*^- ^mutant defects since it is identical to Epb4.1l5^long ^until amino acid 444 after which there are ~60 unique amino acids that end with a predicted PDZ binding domain that is the same class as that in Moe and Epb4.1l5^long^. Injection of mRNA encoding *Epb4.1l5*^*short *^into *moe*^- ^mutants at the 1–4 cell stage failed to rescue *moe*^- ^mutant embryonic defects and failed to apically relocalize ZO-1 and anti-panCrb labeling in the retina or brain (Figure 3K, L). Epb4.1l5^short ^labeling with the Epb4.1l5^short ^specific antibodies appears more cytoplasmic in injected *moe*^- ^mutants (Figure 3J).

Because many proteins with PDZ domains have been implicated in the establishment of cell polarity, we asked whether the PDZ-binding domain in Epb4.1l5^long ^is necessary for its function. We injected mRNA constructs encoding Epb4.1l5^long ^with the PBD deleted (Epb4.1l5^long_ΔPBD^), Epb4.1l5^long ^where its PBD is replaced by PBD from Epb4.1l5^short ^(Epb4.1l5^long+short_PBD^) and Epb4.1l5^short ^where its PBD is replaced by the PBD from Epb4.1l5^long ^(Epb4.1l5^short+long_PBD^). Injection of *epb4.1l5*^*long_ΔPBD *^mRNA failed to rescue Crb2a and ZO-1 apical localization in the retina or rescue brain ventricle formation (Figure 3O, P). However, injection of *epb4.1l5*^*long+short_PBD *^or *epb4.1l5*^*short+long_PBD *^mRNAs did lead to apical relocalization of ZO-1 and anti-panCrb labeling (Figure 3Q-T).

We confirmed that protein was expressed from each of the template rescue mRNAs by western analysis of injected zebrafish. Epb4.1l5^long ^and Epb4.15^short ^were not detectable at time points beyond 72 and 48 hpf respectively (Figure 3U). Both Epb4.1l5^long+short_PBD ^and Epb4.1l5^short+long_PBD ^were expressed at 6 hpf and faint signal was visible at 24 hpf. Neither Epb4.1l5^FERM ^nor Epb4.1l5^long_ΔPBD ^were detectable at time points beyond 6 hpf (Figure 3V), therefore a failure to rescue any *moe*^- ^defects in these cases may be due to a rapid loss of the protein generated by rescue constructs.

### Analysis of rescue with chimeric Epb4.1l5 PBD isoforms

At 60 hpf, mutant *moe*^- ^embryos exhibit reduced or absent brain ventricles, pericardial edema, and RPE defects (Figure 4A, B). Injection of *epb4.1l5*^*short+long_PBD *^mRNA restored RPE integrity (Figure 4C) and retinal lamination (data not shown) in *moe*^- ^mutants, but did not rescue the edema, brain ventricles were small or absent, and the tail curved (Figure 4C, data not shown).

In a heterozygous *moe*^+/- ^incross, a roughly Mendelian inheritance (20/88; 23%) of individuals injected with *epb4.1l5*^*long+short_PBD *^mRNA exhibited RPE defects; however, in all but one case, those defects were minor compared to uninjected *moe*^- ^mutants and their detection required very careful examination (Table 1). The severity of RPE defects varies in uninjected *moe*^- ^mutants: an examination of 35 uninjected *moe*^- ^mutants showed that 8 had mild RPE defects (23%, Figure 4G), 26 had moderate RPE defects (74%, Figure 4I), and 1 had severe RPE defects (3%, Figure 4K). The shift in severity of RPE defects from mostly moderate in *moe*^- ^mutants to nearly normal in injected *moe*^- ^mutants, suggests that injection of *epb4.1l5*^*long+short_PBD *^mRNA largely restores RPE integrity.

Because Epb4.1l5^long+short_PBD ^largely restored RPE integrity in *moe*^- ^mutants we examined whether retinal lamination was also restored in these individuals. At 4 dpf, in wildtypes, GPF^+ ^rods are localized adjacent to the RPE (Figure 4F). In *moe*^- ^mutants, GPF^+ ^rods are ectopically localized throughout the retina, regardless of the severity of RPE defects (Figure 4H, J, L). In *moe*^- ^mutants injected with of *epb4.1l5*^*long+short_PBD *^mRNA, most GPF^+ ^rods localize normally and are adjacent to the RPE. (Figure 4N).

When we injected *epb4.1l5*^*long+short_PBD *^mRNA into embryos resulting from an incross of *moe*^-/+ ^individuals, we observed that more than 25% exhibited edema, suggesting that injection of *epb4.1l5*^*long+short_PBD *^mRNA has a tissue-dependent dominant negative effect. We determined that 61% of embryos had pericardial edema and 96% had small or missing brain ventricles (Table 1). We next injected *epb4.1l5*^*long+short_PBD *^mRNA into embryos from a wildtype incross and found a large proportion of these individuals exhibited pericardial edema and brain ventricle defects, but the RPE was normal, confirming that the dominant negative effects of Epb4.1l5^long+short_PBD^ are limited to edema and brain ventricle formation (Table 1 and Figure 4O). Lastly, Epb4.1l5^long+short_PBD ^retains immunoreactivity with the anti-Epb4.1l5^long ^sera, and we show that the protein localizes cortically like Epb4.1l5^long ^(Figure 4F), suggesting that the Epb4.1l5^long ^PBD is not required for cortical localization.

### Early Epb4.1l5 function rescues later retinal lamination and function

**Figure 5:**
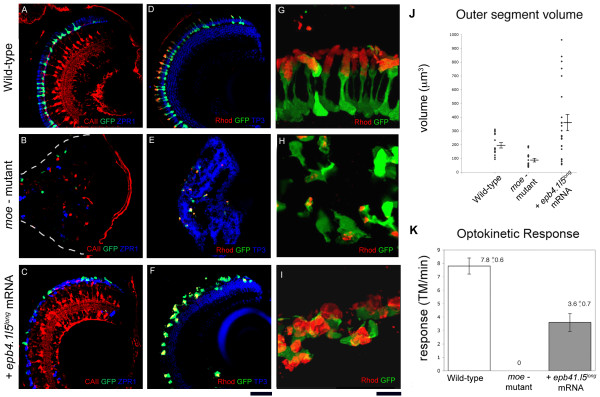
**Injection of *epb4.1l5*^*long *^mRNA into *moe*^- ^mutants restores retinal lamination but not normal photoreceptor morphology at 6 dpf**. In wild-type (A) and *epb4.1l5*^*long *^mRNA injected *moe*^- ^mutants (C), Müller glial cells are radially oriented (labeled with anti-Carbonic Anhydrase II, red), and GFP^+ ^rods and double cones (ZPR-1 antibody, blue) are adjacent to the RPE. (B) In *moe*^- ^mutants, Müller glial cells (red), and rods (green) and double cones (blue) are fewer in number and are localized ectopically. White dashed line indicates the back of the eye. (D) In wild-type larvae, distinct nuclear layers (labeled by TO-Pro3, blue) are visible and outer segments labeled by anti-Rhodopsin (red) are aligned radially and project toward the RPE. (E) In *moe*^- ^mutants, distinct nuclear layers are absent and rod morphology is abnormal. (F) In *epb4.1l5*^*long *^mRNA injected *moe*^- ^mutants, distinct nuclear layers are visible but rod photoreceptor morphology is abnormal. (G, H, I) High magnification confocal z-projections of GFP^+ ^rods labeled with anti-Rhodopsin (red) in wild-type (G), *moe*^- ^mutant (H), and *epb4.1l5*^*long *^mRNA injected *moe*^- ^mutant (I). (J) Outer segment volume at 6 dpf in wildtypes, *moe*^- ^mutants, and *epb4.1l5*^*long *^mRNA injected *moe*^- ^mutants. The average volume of rod outer segments in *epb4.1l5*^*long *^mRNA injected *moe*^- ^mutants is 362 μm^3 ^(+/- 57.1) which was significantly greater than the wild-type volume of 197 μm^3 ^(+/- 18.5), *p *= 0.025. The volume of *moe*^- ^rod outer segments was 90.9 μm^3 ^(+/- 12.3), significantly smaller than wild-type rods (*p *< 0.001). Significance was determined using the Students T-test. (K) Optokinetic response was measured at 5 dpf in wild-types, *moe*^- ^mutants and *epb4.1l5*^*long *^mRNA injected *moe*^- ^mutants. Wild-type larvae exhibited an average of 7.8 (+/- 0.6) tracked movements (TM)/minute, *epb4.1l5*^*long *^mRNA injected *moe*^- ^mutants exhibited a reduced OKR (3.6 (+/- 0.7) TM/minute). *moe*^- ^mutants exhibited no OKR. N = 10 for each group. Scale bars, 40 μm (A-F), 10 μm (G-I). (A-F, single confocal z-sections, G-I, z-projections).

Because mRNA injection could rescue early *moe*^- ^mutant defects, we investigated whether later defects of retinal development were also rescued, in particular, whether differentiated cells acquired their correct laminar position and photoreceptors their normal morphology. We compared the retinas of 6 dpf wildtypes, *moe*^- ^mutants, and *moe*^- ^mutants injected with *moe *or *epb4.1l5*^*long *^mRNA (Figure 5, data not shown). The western blot experiments and immunohistochemistry (data not shown) showed that there is very little, if any, remaining Epb4.1l5^long ^protein after 3 dpf (Figure 3U), the time at which most photoreceptors begin to undergo morphogenesis. In wild-type retinas, nuclei are arranged in distinct layers, Müller glia are radially oriented and project to and contribute to the inner (basal) and outer (apical) limiting membranes of the retina, and rods and double cones display a polarized morphology with their outer segments projecting into the RPE (Figure 5A, D, G). In *moe*^- ^mutants, nuclei do not form distinct layers and the numbers of Müller glia, rods and double cones are reduced and their morphology is abnormal, although interestingly rods do make outer segments (Figure 5B, E, H).

**Figure 6:**
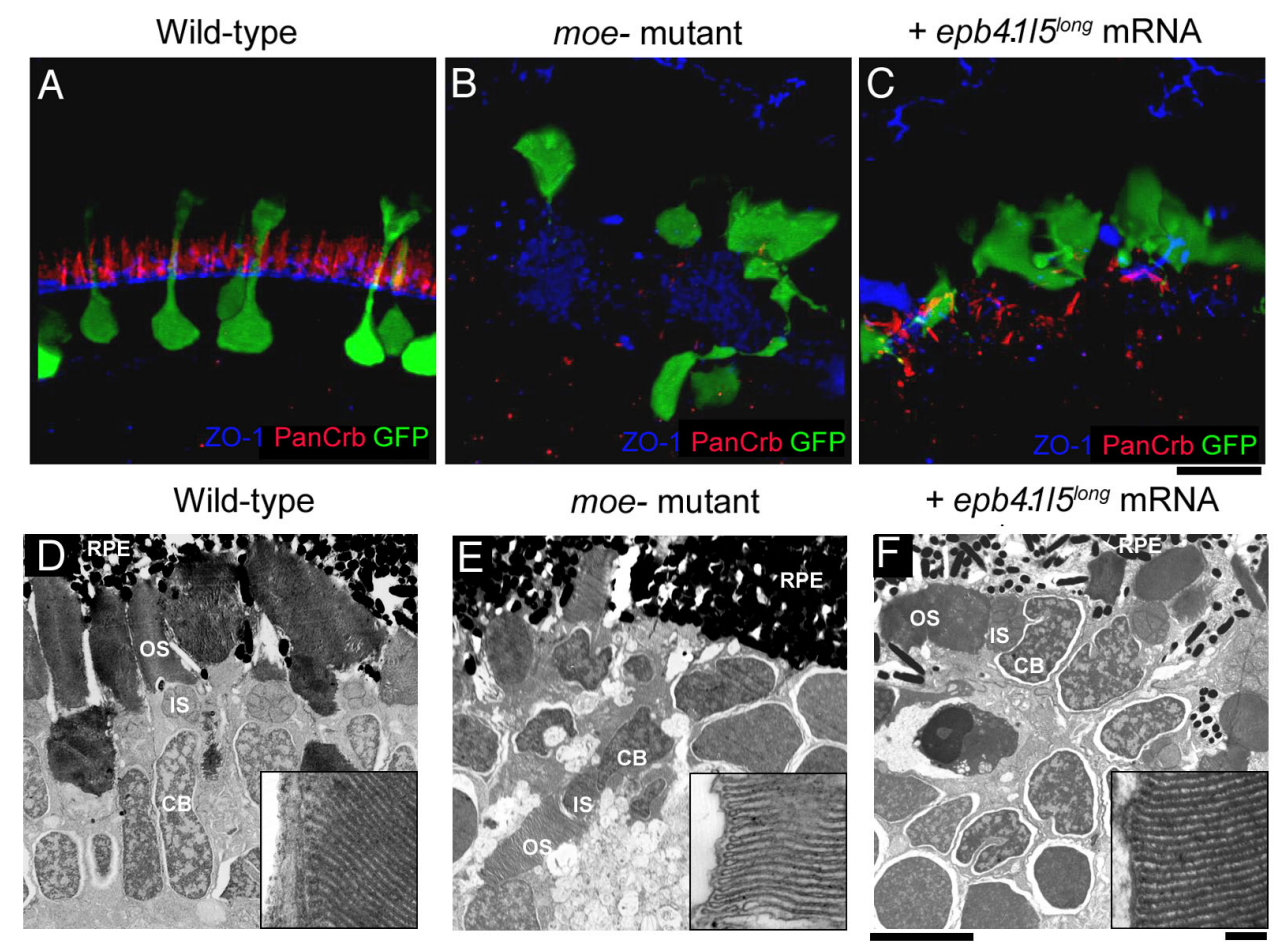
**The outer limiting membrane (OLM) is not restored in *moe*^- ^mutants by injection of *epb4.1l5*^*long *^mRNA**. (A) In wild-type larvae at 6 dpf, Crumbs proteins localize just apical to the OLM, which is labeled by anti-ZO-1. (B) In *moe*^- ^mutants, very little Crumbs protein is visible and ZO-1 labeling is disorganized. (C) In *epb4.1l5*^*long *^mRNA injected *moe*^- ^mutants, streaks of panCrb labeling are visible, but an organized OLM is absent. Ultrastructural transmission electron microscopic analysis at 6 dpf in wild-type (D), *moe*^- ^mutants (E) and *epb4.1l5*^*long *^mRNA injected *moe*^- ^mutants (F) retinas. Electron dense outer segments are seen in all individuals. Insets, higher magnifications of rod outer segments showing regular disc stacking is present in all individuals (100,000X). RPE, retinal pigmented epithelium; OS, outer segments; IS, inner Segments; CB, cell body. (A-C) are confocal z-projections, scale bar 10 μm (D-F). Scale bars, 5 μm (D-F), 100 nm (insets).

Whereas Müller glial morphology and retinal lamination are rescued in *moe*^- ^mutants by injection of either *moe *or *epb4.15*^*long *^mRNA, the morphology of photoreceptors (rods and double cones) is not; instead of standing perpendicular to the normal RPE (Fig. 5A, D, G and Fig. 6A), those in *moe*^- ^mutants injected with either *moe *or *epb4.15*^*long *^mRNA, lie collapsed in a twisted heap adjacent to the RPE (Fig. 5C, F, I and Fig. 6C, and data not shown). The failure to rescue photoreceptor morphology is likely because Moe or Epb4.15^long ^protein from injected mRNA is lost by the time photoreceptors undergo morphogenesis.

Previously, we showed in genetic mosaics that the outer segments of rods lacking *moe *function are larger than those in wild-type rods [21]. We sought to determine whether outer segments are also larger in rods in larvae where all cells have lost *moe *function by about 3 dpf. We measured the size of rod outer segments using anti-Rhodopsin labeling. We found that rods in rescued *moe*^- ^mutants (i.e. injected with *epb4.1l5*^*long *^mRNA) were significantly larger than rods in wild-type retinas at 6 dpf, whereas, rods in *moe*^- ^mutants were significantly smaller than those in wild-type retinas (Figure 5J).

We also tested whether injection of *epb4.1l5*^*long *^mRNA could restore vision to *moe*^- ^mutants as measure by the optokinetic response (OKR). The OKR measures the tracking of the eyes to a moving stimulus [26]. In our study the stimulus consisted of alternating white and black vertical bars moving to the right on a projection screen and we measured tracked eye movements (TM) in response to the stimulus over a minute time period. We measured TM in wild-type, *moe*^- ^mutants, and *epb4.1l5*^*long *^mRNA injected *moe*^- ^mutant larvae immobilized in methylcellulose at 5 dpf. We found that wild-type larvae exhibited an average of 7.8 (+/- 0.6) TM/minute, *moe*^- ^mutants completely lacked TM, and *epb4.1l5 *mRNA injected *moe*^- ^mutant larvae had an average of 3.6 (+/- 0.7) TM/minute (Figure 5K). Thus, even though photoreceptors have morphological defects in *epb4.1l5*^*long *^mRNA injection into *moe*^- ^mutants many of these larvae have functional vision as measured by the optokinetic response.

### Crb2a/b protein localization and outer limiting membrane integrity requires moe function, but rod outer segment disc stacking does not

We recently showed that Moe is an important regulator of Crumbs protein localization in the embryo [21], so we were interested in whether *moe *function is also required at later stages in the retina for Crumbs protein localization. Crb2a and Crb2b are the only Crumbs proteins shown to be expressed by zebrafish photoreceptors [4,21] and the antibodies we use recognize both proteins ([21], and data not shown). The ability to rescue the embryonic defects and retinal lamination with *moe *or *epb4.1l5*^*long *^injection allows us to ask whether the localization of Crb2a/b in photoreceptors also requires *moe *function. The western blot analysis indicates very little, if any, Epb4.1l5^long ^protein remains after 3 dpf in *moe*^- ^mRNA injected individuals, so we can examine retinas in *moe*^- ^mutant larvae where early defects have been rescued but where there is no endogenous Moe and little, if any, exogenous Epb4.1l5^long ^protein after 3 dpf. We examined the localization of Crb2a/b in wild-type, *moe*^- ^mutant, and *epb4.1l5*^*long *^mRNA injected *moe*^- ^mutant larvae at 6 dpf. In the wild-type retina, Crb2a/b localizes just apical to the outer limiting membrane (OLM), which is labeled by anti-ZO-1 antibodies (Figure 6A). In *moe*^- ^mutants, very little Crb2a/b protein is detected in the area surrounding GFP^+ ^rod photoreceptors, ZO-1 labeling is highly disorganized suggesting the OLM has not formed, and there is no spatial relationship between Crb2a/b and ZO-1 (Figure 6B). In *epb4.1l5*^*long *^mRNA injected *moe*^- ^mutants, Crb2a/b labeling is evident, but reduced compared to wild-type retinas, and like *moe*^- ^mutants, ZO-1 labeling is disorganized indicating the absence of the OLM, and we also observed no spatial relationship between Crb2a/b and ZO-1 (Figure 6C).

Although labeling of rods with anti-Rhodopsin antibodies in *moe*^- ^mutant and *epb4.1l5*^*long *^mRNA injected *moe*^- ^mutant larvae suggest that outer segments form (Figure 4D, F, G, I), we wanted to determine whether these outer segments were normal ultrastructurally, in particular, whether disk morphology and packing is normal. We examined outer segments by transmission electron microscopy (TEM) at 6 dpf in wild-type, *moe*^- ^mutant and *epb4.1l5*^*long *^mRNA injected *moe*^- ^mutant larvae. We found that disk morphology and packing appeared relatively normal in photoreceptors in *moe*^- ^mutants and *epb4.1l5*^*long *^mRNA injected *moe*^- ^mutants (Figure 6D-F).

## Discussion

In this study we sought to identify functionally important domains in the orthologous FERM proteins, zebrafish Moe and mouse Epb4.1l5. Our strategy was to use evolution, comparative genomics, and protein engineering to discover regions and sequences necessary for rescue of embryonic and early larval defects in *moe *deficient zebrafish. We first established that injection of wild-type *moe *mRNA into *moe*^- ^embryos rescued all embryonic defects with the exception that mild pericardial edema persisted. In mammalian EST databases there are two major splice isoforms of Epb4.1l5, Epb4.1l5^long ^that is 731 amino acids in length and is similar in length and shares sequence identity with Moe beyond the FERM domain (Figure 1) and Epb4.1l5^short ^that is 504 amino acids. These two isoforms are identical until amino acid 444 and interestingly both contain a predicted PDZ-binding domain at their C' terminus. We raised isoform-specific antibodies and examined expression of the two orthologues by western blot of different mouse tissues. Both antibodies recognized a protein of about 75 kDa in all tissues, which is likely to be non-specific reactivity. Anti-Epb4.1l5^long ^recognized a protein of the expected molecular weight of about 100 kDa in eye, brain, heart, lung, kidney and testis; this expression profile agrees well with recently published immunohistochemical and mRNA expression (in situ hybridization) data in mammalian retinal, brain, and kidney tissues [24,27]. Anti-Epb4.1l5^short ^recognized protein of the expected molecular weight of about 56 kDa in brain, liver, lung, kidney, pancreas, spleen and gut. Not all tissues expressed both isoforms, for instance Epb4.1l5^long ^but not Epb4.1l5^short ^is found in the eye and Epb4.1l5^short ^but not Epb4.1l5^long ^is found in the gut, suggesting that the two isoforms may have non-overlapping functions.

We tested whether either of these Epb4.1l5 isoforms could functionally substitute for Moe during embryonic and early larval development. Injection of mRNA encoding Epb4.1l5^long ^into *moe*^- ^mutants rescues *moe*^- ^mutant defects and leads to the restoration of retinal lamination, RPE integrity, normal brain ventricle morphology, and the apical localization of Crumbs proteins and ZO-1 in the developing retina and brain, similar to injection of *moe *mRNA. Like injection of *moe *mRNA into *moe*^- ^mutants, mild pericardial edema persisted in these *epb4.1l5*^*long *^mRNA injected *moe*^- ^mutants. We found that injection of *epb4.1l5*^*short *^mRNA into *moe*^- ^mutants failed to rescue any phenotypic defects and also failed to relocalize Crumbs proteins and ZO-1 to the apical surface of the retina and brain. Interestingly, while exogenous Epb4.1l5^long ^protein is cortically localized in neuroepithelial cells similar to endogenous Moe protein, Epb4.1l5^short ^is localized cytoplasmically, suggesting that either the PBDs or sequences unique to Epb4.1l5^long ^underlie Epb4.1l5^long ^protein localization.

Although edema was reduced in *moe*^- ^mutants by injection of *moe *or *epb4.1l5*^*long *^mRNA, it was never abolished by mRNA injection. We presume that the edema in *moe*^- ^mutants is caused by kidney dysfunction. There are several possible reasons for failure of mRNA injection to rescue kidney function. It is possible that Moe/Epb4.1l5^long ^function is required for a longer period of time or perhaps continually in the kidney and protein from injected mRNA is not around long enough completely restore kidney function. A second possibility is that the Moe and Epb4.1l5^long ^constructs we used lack the sequence(s) needed to rescue kidney function. If this is the case, then those specific sequence(s) do not seem to reside in Epb4.1l5^short^, since injection of *epb4.1l5*^*short *^mRNA failed to rescue any pericardial edema, and the severity of edema was as severe as uninjected *moe*^- ^mutants. There are, however, many minor splice variants of Moe and Epb4.1l5^long ^in the zebrafish and mammalian EST databases.

Because all the proteins so far identified that interact with Moe and Epb4.1l5 (also known as Ymo1) do so via the FERM domain [20,21], we tested whether expression of a Epb4.1l5 construct encoding the first 346 amino acids (Epb4.1l5^FERM^), which includes the FERM domain, could rescue apical Crumbs proteins and ZO-1 localization or any defects in *moe*^- ^mutants. This construct failed to rescue apical Crumbs protein and ZO-1 localization and any *moe*^- ^defects, however, we could not detect Epb4.1l5^FERM ^protein after 6 hpf, suggesting that this mRNA or protein is unstable. The same result was observed when the PDZ-binding domain in Epb4.1l5^long ^was deleted (Epb4.1l5^long_ΔPBD^); there was no phenotypic rescue in *moe*^- ^mutants and no apical Crumbs protein and ZO-1, and we did not detect Epb4.1l5^long_ΔPBD ^protein after 6 hpf. These observations suggest that the PDZ-binding domain might be important for stability of Moe/Epb4.1l5 protein.

PDZ domains are important mediators of protein interactions and have been shown to be important during the establishment and maintenance of cell polarity [28,29]. We sought to identify the importance of the PDZ-binding domain in Epb4.1l5^long ^by replacing it with the PDZ-domain from Epb4.1l5^short ^to generate the chimeric protein Epb4.1l5^long+short_PBD ^and by replacing the PDZ-domain in Epb4.1l5^short ^with the PDZ-domain from Epb4.1l5^long ^to generate the Epb4.1l5^short+long_PBD ^chimera. We found that injection of *epb4.1l5*^*long+short_PBD *^or *epb4.1l5*^*short+long_PBD*^ mRNA into *moe*^- ^mutants rescued apical localization of Crumbs proteins and ZO-1 in the retinal and brain neuroepithelium, RPE integrity (*epb4.1l5*^*long+short_PBD *^almost complete rescue), and retinal lamination, but did not rescue brain ventricle morphology. Furthermore, injection of *epb4.1l5*^*long+short_PBD *^mRNA into wild-type embryos caused a dominant negative phenotype-brain ventricles were small or failed to form. These observations have revealed important insights into the function of Moe/Epb4.1l5 and suggest that both the PDZ-binding domain of Moe/Epb4.1l5^long ^and additional internal sequences in the C'terminal domain Moe/Epb4.1l5^long ^(aa 444–727) are important for the localization of ZO-1 and Crumbs proteins, RPE integrity and retinal lamination.

Several observations support the importance of both the PDZ-binding domain of Epb4.1l5^long ^and internal sequences in Epb4.1l5^long^. The importance of the PDZ domain in Epb4.1l5^long ^is shown by the experiment where injection into *moe*^- ^mutants of the chimeric construct *epb4.1l5*^*short+long_PBD*^, in which the PDZ-binding domain of Epb4.1l5^short ^is replaced by the PDZ-binding domain from Epb4.1l5^long^, rescues apical ZO-1 and panCrb, retinal lamination and RPE integrity, whereas, injection of *epb4.1l5*^*short *^mRNA does not. This result suggests that there is some specificity between the PBD of Epb4.1l5^long ^and the PBD of Epb4.1l5^short^. The importance of the internal sequence in Epb4.1l5^long ^(aa 444–727) is shown by the experiment where injection into *moe*^- ^mutants of the chimeric construct *epb4.1l5*^*long+short_PBD*^, in which in which the PDZ-binding domain of Epb4.1l5^long ^is replaced by the PDZ-binding domain from Epb4.1l5^short^, rescues apical ZO-1 and panCrb, retinal lamination and mostly RPE integrity, whereas, injection of *epb4.1l5*^*short *^mRNA does not. Unexpectedly, injection of *epb4.1l5*^*long+short_PBD *^mRNA into wild-type embryos caused a dominant phenotype that included brain ventricle defects and edema that are similar to those in *moe*^- ^mutants. One possibility is that Epb4.1l5^long+short_PBD ^competes with endogenous Moe and takes a protein necessary for brain ventricle formation away from the Crumbs complex. Taken together, our experiments have suggested that Moe/Epb4.1l5 proteins are modular proteins and that the PDZ-binding domains have specificity in some tissues.

Moe and other cell polarity determinants, Crb2a/Ome, aPKCλ, and Nok, are required for proper lamination of the zebrafish retina [4-8,22]. The time at which these proteins are needed for lamination has not been determined. Since we see very little Moe or Epb4.1l5^long ^protein after 60 hpf, we suggest that early Moe or Epb4.1l5^long ^function is sufficient to rescue retinal lamination and function. In *moe *or *epb4.1l5*^*long *^injected *moe*^- ^mutants at 6 dpf, Müller glial cell processes are properly oriented and span the thickness of the retina, and the retina has distinct nuclear layers. Furthermore, the vast majority of rod and double cone photoreceptors localize correctly to outer most portion of the retina to form an outer nuclear layer.

Immunohistochemical and ultrastructural analysis of Epb4.1l5^long ^rescued *moe*^- ^mutant retinas, revealed that rescued rods form outer segments, but they were not always oriented with their outer segments toward the RPE (Figure 5D-I, 6D-F). This may be a consequence of the failure of the rescued individuals to establish or maintain the OLM (Figure 6C). Despite the morphological defects of rods and cones in the *moe*^- ^mutants injected with *epb4.1l5*^*long *^mRNA, many of these larvae are visually competent as tested by optokinetic response. Interestingly, our ultrastructural analysis revealed that *moe*^- ^rods formed outer segments, complete with organized membranous discs, suggesting that mechanisms that dictate apical opsin transport and disc formation do not require *moe *function.

Previously, we showed that Moe function is required for the localization of Crb2a and ZO-1 protein at the apical surface of the developing retina in zebrafish embryos [7,21]. We show here that in the wild type retina at 6 dpf, anti-panCrb labeling localizes just above the OLM in the subapical region. In *moe*^- ^photoreceptors, anti-panCrb labeling is not detectible, and ZO-1 appears disorganized. When we examined photoreceptors in *epb4.1l5*^*long *^mRNA rescued *moe- *mutants at 6 dpf, which is several days after detectable Epb4.1l5^long ^protein, we observed that both ZO-1 and Crumbs proteins are present in the photoreceptor region (Figure 6C). The design of our rescue experiment allowed us to analyze the localization of Crumbs proteins and ZO-1 in rescued *moe- *photoreceptors several days after exogenous Epb4.1l5^long ^was gone. In *epb4.1l5*^*long *^mRNA injected *moe- *mutants, ZO-1 and panCrb labeling is not normal and mislocalized ectopic plagues of ZO-1 and panCrb labeling appears to be at the interface of photoreceptors, and/or photoreceptors and Müller glia and there is no clear relationship between ZO-1 and anti-panCrb labeling. Thus, Epb4.1l5/More function is required to maintain, or establish, the OLM and the localization of Crumbs proteins relative to it.

We measured the size of rod outer segments in *moe*^- ^mutants that had been injected with *epb4.1l5*^*long *^mRNA but that lack measurable Moe protein during photoreceptor morphogenesis, and found that these genetically *moe*-deficient rods were nearly twice the normal size (362 μm^3 ^compared to wild-type outer segments 197 μm^3^). This observation is in agreement with previous data from our lab and others implicating Moe and the Drosophila orthologue, Yurt, as negative regulators of apical membrane size in photoreceptors [20,21]. We observed that in uninjected *moe- *mutants, rod outer segments are smaller than wild-types (90.9 μm^3 ^compared to 362 μm^3^), this could be a consequence of the general ill health of *moe *mutants at 6 dpf, and/or the isolation of photoreceptors from factors secreted by the RPE and Müller glia [30,31].

## Conclusion

Our strategy to use comparative genomics and protein engineering has revealed that the function of Moe/Epb4.1l5 protein is modular and that particular regions can be assigned particular functions. We also show that the C' terminal domain that encodes the PDZ-binding domain in Moe and Epb4.1l5^long ^is important but not sufficient to confer full protein function to the FERM domain and that other sequences in Moe and Epb4.1l5^long ^are important. The next challenge will be to identify the PDZ-containing protein that interacts with the PDZ-binding domain in Moe/Epb4.1l5^long ^and the additional protein(s) that interact with the unique sequences in Epb4.1l5^long^. Although the role of the Crumbs complex in epithelial morphogenesis has been much studied, the molecular mechanism of the Crumbs complex function is still unknown. The identification of additional proteins that interact with Moe/Epb4.1l5^long ^may help to determine the mechanistic function of the Crumbs complex.

## Methods

### Animals

AB wild-type strain and the *moe*^*b*781 ^allele were maintained and staged as described previously [7,32]. For analysis requiring EGFP-expressing rods, the *moe*^*b*781 ^allele was crossed into the *Tg(Xop:EGFP) *transgenic line [33]. To block pigmentation, embryos were treated with 2.5 μg/mL phenylthiourea (PTU) beginning at about 20 hpf.

### RNA injections

For mRNA transcription, PCRTopoII or pBSII vectors containing the cDNAs of full-length *moe*, *epb4.1l5*^*long*^, *epb4.1l5*^*short*^, or myc-tagged fusions of the following constructs; *epb4.1l5*^*long*^, *epb4.1l5*^*FERM *^(1–346 N-terminal amino acids), *epb4.1l51*^*long_ΔPBD*^(epb4.1l5^long ^with the four C'terminal TTEL deleted), *epb4.1l51*^*short+long_PBD *^(short C-terminal AAMTEI replaced with LLTTEL) or epb4.1l5^long+short_PBD ^(long C-terminal LLTTEL replaced with AAMTEI), were linearized by NotI or PspOMI restriction digest, and transcribed with the Sp6 or T7 Message Machine transcription kit (Ambion). Roughly 100–250 pg (amount varied per construct for a final 0.2 fM) of mRNA was injected into yolk of 1–4 cell embryos obtained from an incross of *moe*^*b*781 ^or *moe*^*b*781^/Xop-GFP heterozygotes. For those mRNAs that failed to rescue at the above molarity, we tested whether injecting more rescued, and in all cases higher amounts failed. Concurrent with immunohistochemical analysis, we probed with anti-Moe on alternate sections to confirm the *moe*^- ^genotype. All constructs were sequenced prior to mRNA synthesis.

### Antibody production

We generated rabbit polyclonal antibodies against the C-terminal 20 amino acids of mammalian Crb2 (N' AGARLEMDSVLKVPPEERLI C'; 95% identity with *D. rerio *Crb2a, 90% identity with Crb2b) conjugated to keyhole limpet hemocyanin. This anti-Crb antibody recognizes GST-tagged recombinant purified intracellular domains of *D. rerio *Crb1, Crb2a, Crb2b, Crb3a, and Crb3b by western blot but not GST alone (data not shown). Rabbit antibodies were generated to be specific for either Epb4.1l5^long ^or Epb4.1l5^short ^by immunizing rabbits with purified recombinant His-Epb4.1l5^long_445–731 ^and His-Epb4.1l5^short_445–504 ^protein that was purified with HisLink resin (Promega). All rabbits were immunized and boosted with about 500 μg protein in PBS (University of Massachusetts-Amherst antibody facility). Anti-Epb4.1l5^short ^serum was affinity purified against 2 mg GST-Epb4.1l5^short_445–504 ^(cDNA cloned into pGEX-4T1 vector (Amhersham Biosciences) that was purified with Glutathione Sepharose (Amersham Biosciences), cross-linked to a NHS-activated Sepharose (Amersham Biosciences) column, and eluted in 100 mM glycine pH 2.5. Affinity purified antibodies were dialyzed against PBS, and BSA was added to 1mg/ml and glycerol to 50%.

### Western blot analysis

Protein was extracted from mouse tissues by homogenization in PBS with protease inhibitors (Complete Mini, Roche), 1 mM AEBSF, 0.2 mM Na_2_SO_4_, 1% Trition X-100. 60 μg of total protein from each tissue was resolved on a 10% SDS-PAGE gel. Zebrafish protein was prepared by homogenizing zebrafish embryos or larvae in 2 μl reducing sample buffer (plus protease inhibitors) per individual. To reduce interference from yolk proteins, embryos were deyolked according to Link et al., 2006 [34]. Samples were denatured at 100°C for 5 min, vortexed, and insoluble debris pelleted by centrifugation. Western blotting was conducted according to standard procedures. Primary antibodies: rabbit anti-Epb4.1l5^long ^1:750; affinity purified rabbit anti-Epb4.1l5^short ^1:15, 5 μg/mL final; rabbit anti-Myc 1:10,000 (Bethyl); mouse anti-α-Tubulin 1:2000 (Developmental Studies Hybridoma Bank).

### Immunohistochemistry

Zebrafish embryos and larvae were fixed in 4% paraformaldehyde/0.1M NaPO_4 _pH 7.2 for 1 hr at room temperature, washed in 0.1M NaPO_4 _pH 7.2, embedded in agar, and equilibrated in 30% sucrose, and frozen sections cut (18 μm or 30 μm). Sections were incubated in blocking solution (20% goat serum, 10 mg/mL BSA, 145 mM NaCl, 10 mM lysine, 50 mM Tris pH 7.4, 0.1% Tween-20), then in primary and secondary antibody, overnight at 4°C and 3 hours at room temperature respectively (rabbit anti-Moe 1:500, rabbit anti-panCrb 1:1000, mouse anti-ZO-1 1:10, rabbit anti-CAII 1:500, mouse anti-ZPR1 1:100, mouse anti-Rhodopsin 1:100 (B6–30), goat anti-mouse and goat anti-rabbit 1:100 (Molecular Probes and Jackson ImmunoResearch), and TO-PRO-3 1:500 (Invitrogen)). Slides were cover-slipped with Prolong anti-fade reagent (Invitrogen). Images were acquired on a Zeiss 510C META confocal microscope and processed with ImageJ 1.37v [35]. Volume determination of outer segments was performed on albino larvae by quantifying the voxels represented by Rhodopsin (B6–30) labeling in 3D reconstructions with the ImageJ plugin Sync Measure 3D [36].

### Transmission electron microscopy

Larvae were fixed at 6 days in 4% PFA/0.5% gluaraldehyde/0.1M NaPO_4 _pH 7.2 overnight at 4°C. Samples were rinsed, incubated in 1% osmium tetroxide in PBS at room temperature, dehydrated in an ethanol series, and then rotated in a 1:1 ratio of 80% ethanol:LR White resin (Electron Microscopy Sciences) for one hour at room temperature, and in a change of the same solution overnight at room temperature. Samples were then equilibrated in 100% LR White 2X for 2 hours, and polymerized. Ultrathin sections (100 nm) were cut then stained with 2% uranyl acetate for 30 min and 0.5% lead citrate for 12 min at room temperature. Sections were visualized on a JOEL100S Transmission Electron Microscope.

### Optokinetic response

Briefly, Optokinetic Response (OKR) was measured according to protocol modified from Rinner et al., 2005 [37], at 5 dpf in WT, *moe*^b781^, and *epb4.1l5*^*long *^mRNA injected *moe*^b781 ^mutants that were immobilized in a drop of 2% Methyl Cellulose. A dissecting scope was used to visualize Tracked eye Movements (TM) per minute in response to a moving visual stimulus. Cycling vertical lines were generated with Optomotor 1.2 software (*f*, 1Hz; λ, 120 pxls; speed, 5), and rear projected with an inFocus LCD digital projector on a 180° curved screen approximately 4.5 cm from larvae (Optomotor 1.2 software was generously provided by Harold Burgess).

## Authors' contributions

AKC participated in study design, data collection and analysis, and drafted the manuscript. AMJ conceived of the study, and participated in its design and drafted the manuscript.
